# Investigation of a Canine Parvovirus Outbreak using Next Generation Sequencing

**DOI:** 10.1038/s41598-017-10254-9

**Published:** 2017-08-29

**Authors:** Jayme Parker, Molly Murphy, Karsten Hueffer, Jack Chen

**Affiliations:** 1 Department of Biology and Wildlife, Institute of Arctic Biology, University of Alaska Fairbanks, Fairbanks, AK 99775 USA; 2 Alaska State Public Health Virology Laboratory, Fairbanks, AK 99775 USA; 3 Department of Veterinary Medicine, University of Alaska Fairbanks, Fairbanks, AK 99775 USA

## Abstract

Canine parvovirus (CPV) outbreaks can have a devastating effect in communities with dense dog populations. The interior region of Alaska experienced a CPV outbreak in the winter of 2016 leading to the further investigation of the virus due to reports of increased morbidity and mortality occurring at dog mushing kennels in the area. Twelve rectal-swab specimens from dogs displaying clinical signs consistent with parvoviral-associated disease were processed using next-generation sequencing (NGS) methodologies by targeting RNA transcripts, and therefore detecting only replicating virus. All twelve specimens demonstrated the presence of the CPV transcriptome, with read depths ranging from 2.2X – 12,381X, genome coverage ranging from 44.8–96.5%, and representation of CPV sequencing reads to those of the metagenome background ranging from 0.0015–6.7%. Using the data generated by NGS, the presence of newly evolved, yet known, strains of both CPV-2a and CPV-2b were identified and grouped geographically. Deep-sequencing data provided additional diagnostic information in terms of investigating novel CPV in this outbreak. NGS data in addition to limited serological data provided strong diagnostic evidence that this outbreak most likely arose from unvaccinated or under-vaccinated canines, not from a novel CPV strain incapable of being neutralized by current vaccination efforts.

## Introduction

Canine parvovirus type 2 (CPV-2) is a non-enveloped, single-stranded DNA virus that causes fatal gastroenteritis in young dogs^1^. The CPV-2 genome is 5323 nucleotide long and possesses at least 2 major open reading frames (ORFs)^2^. CPV-2 infections are associated with significant morbidity and mortality which can reach 91% in untreated pups^3^. Three variants of CPV type 2 are known, CPV-2a, CPV-2b, and CPV-2c, and are highly contagious due to suspected low infective dose requirements combined with high titers of transmissible virus in stools of affected dogs^4, 5^. The virus is highly resistant to environmental conditions and can remain viable outside of its host for at least a year^6^.

CPV-2 infections are one of the most common causes of disease outbreaks in dense canine environments such as kennels or shelters, and timely diagnosis is important in order to control the number of affected individuals^7^. CPV is commonly diagnosed in veterinary clinics using rapid fecal enzyme-linked immunosorbent assays (ELISA) that target viral antigen. These tests have high specificity but poor sensitivity when compared to PCR or immune-electron microscopy^8^. Although PCR assays are more sensitive, they can cause difficulty in terms of result interpretation since they can detect live attenuated vaccine strains or produce positive results from dogs showing no symptoms of gastroenteritis. This requires veterinarians to associate PCR results with other clinical signs of CPV infection, the animal’s history, and other laboratory parameters such as leukopenia^8, 9^. The performance of any of these antigen-targeting methods are highly variable due to the known phenomenon of intermittent shedding of CPV during the earlier and later stages of disease^10^. Despite these diagnostic challenges, it has been shown that current ELISA and PCR methods are capable of detecting all three variants of CPV in spite of antigenic differences^11^.

Between January and April 2016, the interior of Alaska experienced an increased number of CPV cases^12–14^. Outbreaks of canine infectious disease in Alaska can be socially and economically detrimental due to the dense dog population needed to support the state sport, dog mushing. The interior of Alaska, in particular, has a concentrated population of dogs due to the increased presence of professional and recreational mushers operating various sized kennels of 10–100+ sled dogs each. Each winter, Alaska hosts many visiting mushers, national and international, who come to the area to train and race their own dogs. Additionally, Alaska hosts several high-profile international dog mushing races, including the Iditarod and the Yukon Quest. These large races frequently include groups of dogs ranging from 350–1350 animals in number, who utilize the same trails, rests stops and parking areas, allowing for extensive comingling and a high potential for disease transmission. Sick dogs can spread the virus through defecation on common trails, where other teams run and transport the virus back to their own kennels. In 2016, the interior of Alaska experienced a mild winter with less than the usual amount of snowfall, leading to accumulation of uncovered fecal material on common mushing trails. In response to the CPV-associated outbreak of disease, mushers were provided notifications throughout the 2016 race season, and were asked to not bring potentially infected dogs to races in order to help slow the progression of the outbreak. Recommendations were also made to isolate sick animals in individual kennels. Due to the perceived increased virulence of the CPV strain or strains associated with the outbreak, additional testing to further characterize the virus was pursued.

Sequence analysis of the VP2 gene, the most abundant and immunogenic protein produced for construction of the viral particle capsid, is used to help subtype and further characterize wildtype CPV^15–18^. Surveillance of this particular protein is critical for assessing the potential efficacy of the current vaccination strategy and can also be used to relate individual infections in outbreak situations^19–22^. Beyond VP2 investigation, deep-sequencing of the whole genome has been shown to be useful in order to better understand the true nature of CPV molecular diversity and discover new variants^23, 24^.

As a clinical laboratory, it is our approach to target RNA molecules upon initial assessment of a clinical specimen to account for common RNA viruses as well as DNA viruses being actively transcribed while simultaneously reducing background genomic DNAs. In this experiment, we use next-generation sequencing (NGS) to detect and characterize actively replicating CPV in rectal swabs of canines associated with a suspected outbreak in the interior of Alaska between January - April 2016 by targeting RNA transcripts.

## Materials and Methods

### Specimens

Twelve rectal swab specimens representing two communities, A (n = 5) & B (n = 7), were collected for the sole purpose of disease diagnosis. Communities involved are 258 kilometers (approximately 160 miles) apart. The University of Alaska Fairbanks Institutional Animal Care and Use Committee (IACUC) has determined that this research project did not require IACUC review. Protocol review is not required for diagnostics performed during the course of a disease investigation. All methods were carried out in accordance with relevant guidelines and regulations.

### Referral Testing (serology, PCR, and genotyping)

Specimens were sent to Cornell University, Ithaca, NY for evaluation of CPV IgG and IgM antibodies using hemagglutination inhibition (HAI), nucleic acid using PCR, and genotyping (Table 1). Total antibody was evaluated, as well as the IgM to IgG ratio upon application of 2-mercaptoethanol to dissociate IgM antibody molecules. Laboratory interpretation guidelines suggest that a 4-fold or greater decrease in titer after 2-mercaptoethanol treatment is evidence of recent parvovirus exposure. Post-vaccination levels of total antibody can range from 80 to 2,560 HAI, with ranges around 80 demonstrating need for booster vaccination. In addition to serotyping, canine parvovirus PCR was used to rule-in specimens containing CPV nucleic acid and two specimens were genotyped.

**Table 1:** Specimens and Referral Results Summary.

Sample ID	Description	Community (A or B)	Total Ab (HAI)	IgG only (HAI)	CPV PCR	Genotyping
Parvo1	8 wk old	A	—	—	Positive	2/2a
Parvo2	Same animal as Parvo1 (reproducibility specimen)	A	—	—	Positive	2/2a
Parvo3	8 wk old littermate to Parvo1/2	A	—	—	Positive	—
Parvo4	6 mo. old	B	—	—	Positive	—
Parvo5	1.5 yr. old	B	—	—	Positive	—
Parvo6	2 yr. old, moved from affected Community B to Community A	A	160	10	Positive	—
Parvo7	6 yr. old, next door neighbor to Parvo6. Died within one day of Parvo6.	A	80	<10	Positive	—
Parvo8	6 mo. old	B	—	—	—	—
Parvo9	Unknown	B	—	—	—	—
Parvo10	5 mo. old	B	—	—	—	2b
Parvo11	10 yr. old	B	—	—	Positive	—
Parvo12	20 mo. old	B	1,280	<40	Positive	—

— = Not Tested, HAI = hemagglutination inhibition.

### Nucleic acid isolation in preparation for sequencing

Flocked swabs were used to collect specimens from the rectum of expired canines presumed to be related to the outbreak (n = 12). Confirmation of parvoviral infection was made by rapid fecal enzyme-linked immunosorbent assays (ELISA), necropsy findings consistent with acute parvoviral illness (hemorrhagic enteritis with fibrinous serositis), or both. Swabs were immediately placed in viral transport medium to stabilize viral particles. Representative aliquots (~1 mL) of each specimen were centrifuged for 10 minutes at 15,000 rpm to pellet and discard debris as well as the majority of the host and bacterial cells. The supernatant was transferred into a new centrifuge tube and a portion of the supernatant (~500uL) was used as the starting material for nucleic acid isolation using phenol/chloroform followed by ethanol precipitation as previously described^25, 26^. DNAse I (New England Biolabs, Inc.) was added to the isolated total nucleic acid and purified again using phenol/chloroform followed by ethanol precipitation to reduce the overall contaminating genomic DNAs.

### Library preparation for sequencing

NEBNext Ultra RNA Library Prep Kit for Illumina (New England Biolabs, Inc) was used to construct sequencing library from a starting quantity of 10–200ng of total RNA. Individual indexes were used to barcode the fragments and allow for specimen pooling. Fragment sizes of ~300 bp and larger were selected during AMPure bead cleanup.

### Sequencing and analysis

Libraries were pooled and sequenced using the Illumina MiSeq system and the MiSeq Reagent Kit v2 (Illumina) 500-cycle sequencing kit. Paired-end sequencing was performed (2 × 251 bp) to achieve all base reads available in the 500-cycle sequencing format. Read files generated by the sequencer were analyzed by GSNAP reference sequence alignment using NCBI Accession NC_001539.1 as the reference genome for parvovirus.

## Results

Serological data was difficult to obtain since affected animals would expire before blood draws could be acquired. Only three dogs in this data set were tested for canine parvovirus antibodies (Parvo6, 7, and 12) each demonstrating low to medium levels of acceptable antibody (Table 1). PCR was performed on most specimens in this dataset unanimously ruling-in the presence of CPV nucleic acid. Three specimens were genotyped, Parvo1 and Parvo2 (representing the same animal and therefore an opportunity to assess diagnostic reproducibility) were typed as a 2/2a virus, and Parvo10 as a 2b virus.

Table 2 describes the general sequencing metrics for each specimen tested. The number of reads aligning to the canine parvovirus reference sequence (NC_001539.1) ranged from 47 reads for Parvo11 to 263,625 reads for Parvo9. This type of range can be expected when blindly testing clinical specimens and is dependent on sample collection and handling as well as the stage of disease presenting in the canine at the time of specimen collection. Read depth ranged from 2.2X to 12,381X and genome coverage ranged from 44.8% to 96.5% when aligned to the reference genome. Canine parvovirus sequences were heavily masked amongst other sequence data representing an average of 0.95% (range 0.0015% to 6.7%) of the metagenome across all specimens sequenced.

**Table 2:** NGS metrics after aligning read files to reference genome NC_001539.1 (canine parvovirus).

Sample ID	Sequencing Output (Megabases)	Total Reads	Number of Viral Reads Aligned to Ref. genome	Viral Reads of Total Reads (%)	Read Depth (X)	Genome Coverage (%)
Parvo1	437.3	1,749,240	277	0.02	13.0	87.8
Parvo2	418.4	1,673,478	1,114	0.07	52.3	93.4
Parvo3	500.0	1,999,824	10,450	0.52	490.8	94.8
Parvo4	924.6	3,698,580	2,418	0.07	113.6	92.4
Parvo5	582.8	2,331,346	44,288	1.90	2,080.0	95.1
Parvo6	630.9	2,523,606	29,789	1.18	1,399.1	96.5
Parvo7	585.3	2,341,324	7,543	0.32	354.3	93.1
Parvo8	509.6	2,038,224	2,744	0.13	128.9	89.1
Parvo9	977.5	3,909,948	263,625	6.74	12,381.4	94.9
Parvo10	348.2	1,392,626	2,906	0.21	136.5	89.4
Parvo11	767.3	3,069,074	47	0.0015	2.2	44.8
Parvo12	435.3	1,741,200	3,439	0.20	161.5	93.5

**Figure 1:**
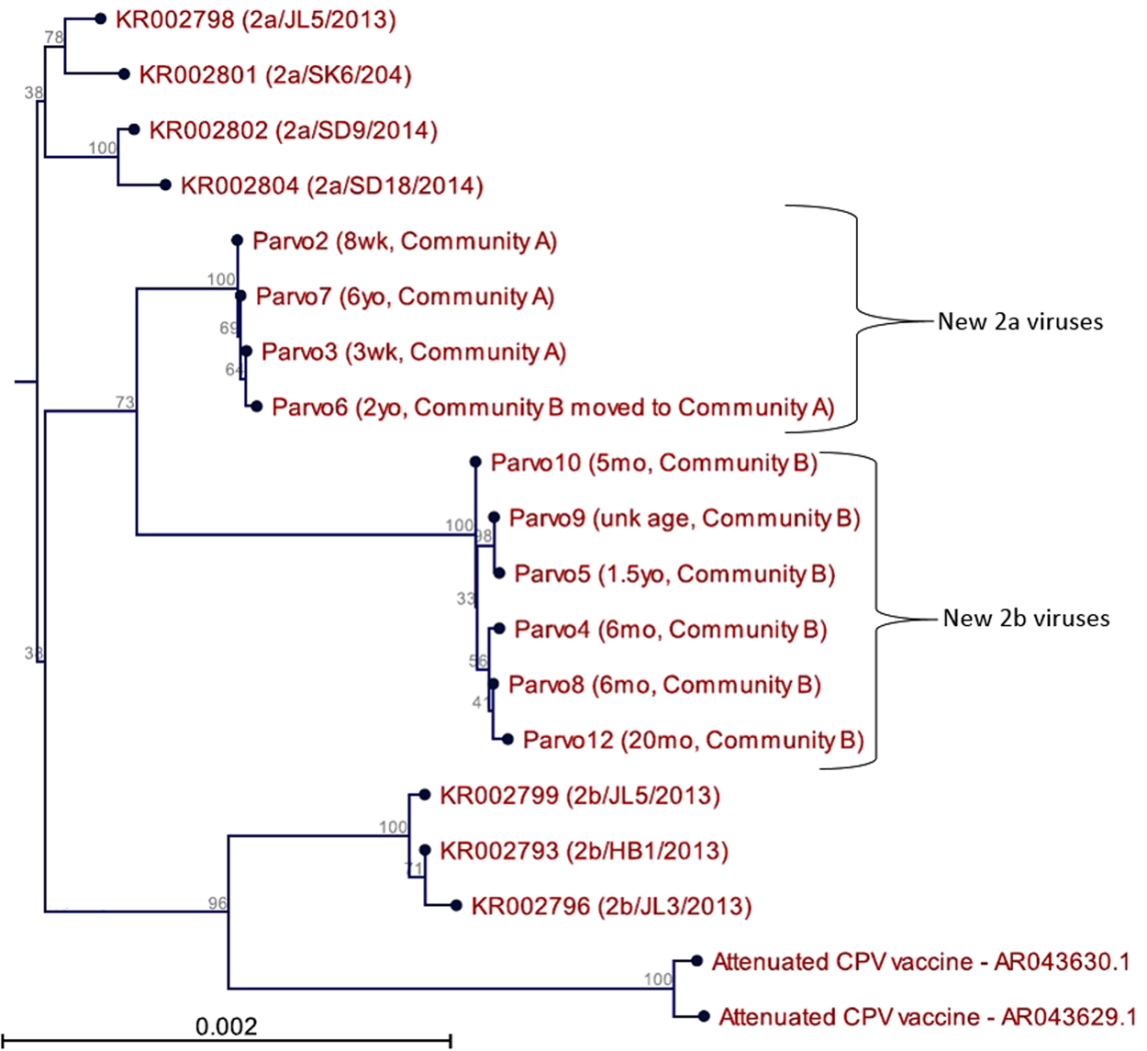
Phylogram of clinical specimen isolates in relation to reference sequences of canine parvovirus 2a, 2b, and vaccine candidate genomes. Parentheses next to 10 of the 12 specimens analyzed indicate the age of the canine and kennel origination. Two specimens, Parvo1 and Parvo11, are not included in this analysis due to inadequate read depth and subsequent insufficient sequence availability. Two major groupings are recognized as distinctly 2a viruses (Parvo2, 7, 3, 6) and 2b viruses (Parvo10, 9, 5, 4, 8, 12).

Whole genome phylogenetic analysis was performed using CLC Workbench 8 (Fig. 1). Parvo1 and Parvo11 samples were not included in this analysis due their lower than optimum read depths (<30X). Whole genome sequences from the remaining 10 specimens were compared to 13 reference genomes from NCBI representing many variations of subtype 2a and 2b canine parvoviruses as well as two versions of the attenuated vaccine strain. Phylogenetic analysis shows that there were two distinct groups of viruses circulating in the outbreak and these groups were directly associated with the geographical location of the animal when it became ill. Parvo2, 3, 6, &7 formed a group of somewhat older origin and represented animals from Community A. The other grouping of viruses sequenced (Parvo4, 5, 8, 9, 10, & 12) shows more recent evolution and were collected from animals approximately 260 kilometers away in Community B. Interestingly, Parvo6 was a dog that had been transferred from Community B to Community A; however, the sequence data would suggest that the parvovirus infection was actually caused by a virus picked up in Community A, not brought in from Community B.

**Figure 2:**
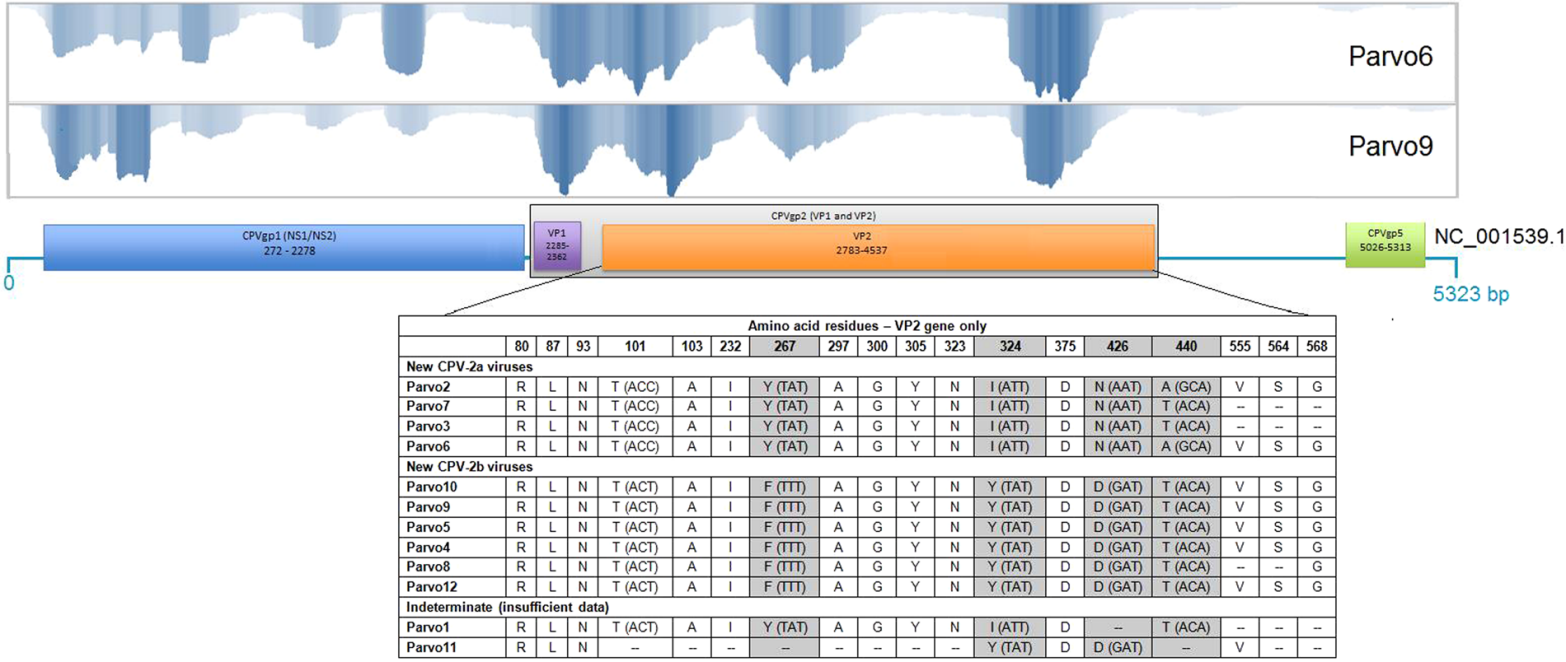
Visual depiction of sequence alignment to reference genome, VP2 gene location within the canine parvovirus genome and analysis of 18 amino acid positions. Parvo6 and Parvo9 were chosen as representatives of each group of viruses to visualize NGS data and varying sequencing depths for each major protein. The VP2 region of each specimen was analyzed at key amino acid positions reflecting canine parvovirus subtype. Parvovirus specimens are ordered and categorized as they are depicted in Fig. 1 (phylogram). “New” canine parvovirus 2a and 2b stem from emerging viruses showing variability at the 426 aa position^27^.

Further analysis of the VP2 protein of each one of these groupings was performed (Fig. 2). Parvo6 represented the Community A grouping and Parvo9 represented the Community B grouping due to their high genome coverage percentages and read depths (>30X). The nucleic acid VP2 sequences for these two viruses were 99.4% similar differing by 10 bases. Five (50%) of the base differences resulted in four amino acid sequence differences in the VP2 protein at residues 267, 324, 426, and 440. The amino acid difference at residue 426 is a common codon affected by polymorphism and is used to diagnostically differentiate CPV-2a and CPV-2b viruses with the use of specific sequence-based probes. The amino acid difference at position 426 for Parvo6 was asparagine, specific to CPV-2a viruses, and for Parvo9 was aspartic acid which is specific to CPV-2b viruses. The amino acid residue at position 297 was alanine for both Parvo6 and Parvo9 suggesting they are new strains of CPV-2a and −2b, as described by Decaro *et al*.^27^.

## Discussion

The NGS methodology proved to be an effective diagnostic tool for further characterizing CPV in rectal swabs of affected canines. Not only was the virus detected, but the RNA for all viral proteins was obtained in many cases suggesting that the virus was undergoing active replication and causing symptoms. Despite only targeting RNA transcripts, near whole genomes were obtained making it possible to construct a phylogenetic tree that demonstrated the presence of 2 distinct subtypes of a CPV virus, CPV-2a present in dogs living in and around Community A and CPV-2b present in dogs living in Community B. Similarity of these 2 CPV virus groups to previously published CPV sequences as well as the limited presence of protective CPV antibodies in tested serum suggest that the outbreak did not involve a novel strain of CPV, but rather exposure in an under-vaccinated on unvaccinated, and therefore immunologically naive, dog population.

### Summary Line

Next-generation sequencing was used for diagnosing and further characterizing a canine parvovirus outbreak in Alaska.
